# Precision grip control while walking down a step in children with unilateral cerebral palsy

**DOI:** 10.1371/journal.pone.0191684

**Published:** 2018-02-01

**Authors:** Daniela Ebner-Karestinos, Benoît Flament, Carlyne Arnould, Jean-Louis Thonnard, Yannick Bleyenheuft

**Affiliations:** 1 Institute of Neuroscience (IoNS), Université catholique de Louvain, Brussels, Belgium; 2 Physical and Occupational Therapy Departments, Paramedical Category, Haute Ecole Louvain en Hainaut, Montignies-sur-Sambre, Belgium; 3 Physical and Rehabilitation Medicine Department, Cliniques Universitaires Saint-Luc, Brussels, Belgium; University of Chicago, UNITED STATES

## Abstract

**Aim:**

To compare grip force (GF) and load force (LF) coordination while walking down a step between children with unilateral cerebral palsy (UCP) and typically developing (TD) children.

**Methods:**

Twenty-five children with UCP (age 9.3±1.7 y) and 25 TD controls (age 9.4±2.1 y) walked down a step while holding a grip-lift manipulandum. Dynamic and temporal variables were analyzed. The maximum voluntary contraction (MVC) was also assessed.

**Results:**

The temporal course was perturbed mainly in the more affected hand of children with UCP when compared to TD children because the increases in GF and LF onset occurred in a reversed order. Compared with the TD controls, the children with UCP presented higher LF values on both hands and a higher GF on the less affected hand. In children with UCP, the GF to LF adaptation was adequate on the less affected hand but overestimated on the more affected hand. Furthermore, children with UCP presented a lower MVC in the more affected hand, leading to a higher percentage of MVC used during the task.

**Interpretation:**

Our findings highlight an anticipatory control of precision grip during a stepping down task in children with UCP that is adequate for the less affected hand but altered for the more affected hand.

## Introduction

Children with unilateral cerebral palsy (UCP) present one-sided motor impairments. They are usually able to walk independently [1], but have difficulties performing daily activities with their upper extremities [2]. Children with UCP generally have impairments in their precision grip, which is needed to pick up small objects between the thumb and index finger [2–4].

Precision grip requires good coordination between the grip force (GF), which is perpendicular to the object’s grip surface, and the tangential load force (LF). It keeps the object from slipping and prevents early muscle fatigue [5]. This coordination results from: 1) predictions of movement that allow a person to generate an adequate GF based on sensorimotor information (feedforward system) [6–10], and 2) the ability to update these predictions through reactive mechanisms that allow for correction based on actual movements (feedback system) [6, 10–13]. This coordination is not innate [5, 7]. In typically developing (TD) infants, fingertip force coordination relies mainly on reactive mechanisms [5], which develop during childhood to a predictive mechanism that is considered mature at age 6–8 years [5, 14]. Children with UCP present a lack of coordination between LF and GF and display a precision grip pattern of movement similar to that of young TD children around the age of 2 with immature prehension [3, 4, 13–19].

In addition to the adaptations required to regulate GF and LF coordination during smooth movements, specific mechanisms are required when a brisk change in load is generated (e.g., a brisk collision on a handheld object in a sitting position). In such situations, adults [6] and TD children generate an increase in GF that anticipates the brisk increase in load. This increased GF reaches a maximal value after the brisk change, allowing the collision to be dampened and the grasp to be secured, consecutively. When object manipulation is perturbed by a brisk load increase generated by the drop of a mass, children with UCP are able to anticipate this perturbation with their less affected hand, but display impaired anticipation with their more affected hand [20]. The delay between impact to maximal GF is longer and more variable for the more affected hand of children with UCP, suggesting deficits in the anticipation of such dynamic perturbations [20].

In everyday life, brisk load changes are more likely to be induced by the lower extremities than while in a seated position, as reported by previous studies [21–23] and thus, it is likely that smooth movements needed to adapt precision grip become more challenging due to an increase in the degrees of freedom [24]. Load changes could be generated on the upper extremities either in a dynamic cyclic situation such as walking while transporting an object or in dynamic discrete events such as stepping over an obstacle or descending a single step while transporting an object. Prabhu et al. (2011) [21] investigated the coordination of fingertip forces while walking. In typically developing individuals, the GF was well timed to the LF and modulated in a sinusoidal way due to gait-related events. In children with UCP they observed a difference between hands, suggesting the presence of impairments in the coordination of the forces associated with the grasp and the locomotion in the more affected hand. In a discrete task of walking down a step, healthy adults are able to adapt their precision grip to a brisk load change induced by the lower extremities by regulating the forces providing an ideal GF to dampen the LF increase and secure the object in hand [25]. It is unknown whether such adaptation of fingertip forces is present in the more or less affected hand of children with UCP while a brisk load increase is induced on a handheld object by walking down a step. This is of interest due to the amount of everyday life activities that are performed requiring the combined use of upper and lower extremities, such as picking food from a fridge, picking objects from the ground or getting into/out of a car.

The aim of this study was to assess the grip-lift coupling in children with UCP during an intersegmental discrete task of walking down a step with a handheld object. These results were compared with those from a cohort of TD children. We hypothesized that children with UCP would be able to manage the task, but that anticipatory motor control would be impaired for the more affected hand, whereas it would be preserved in the less affected hand.

## Methods

### Participants

A total of 50 children participated in this study, including 25 children with UCP (mean age 9 years 3 months (SD = 1 year 8 months; range 6–12 years; 17 girls). They presented either a right (n = 15) or a left (n = 10) hemiparesis and were classified as levels I (n = 3), II (n = 21) or III (n = 1) according to the Manual Ability Classification System [26], and as levels I (n = 13) or II (n = 12) on the Gross Motor Function Classification System [27]. They were contacted from a cerebral palsy (CP) reference center and had participated or were interested in participating in an intensive intervention study [28]. Children were included if they met the following criteria: (a) age 6 to 13 years old, (b) were able to walk with a handheld object without dropping it, (c) were at a school level equal to that of their TD peers, (d) were able to follow instructions, and (e) had a documented lower extremity impairment in a medical examination. Children were excluded if they: (a) presented uncontrolled seizures, (b) had botulinum toxin injections or orthopedic surgery within the previous 12 months or planned to undergo either procedure within the study period, or (c) had visual problems likely to interfere with testing. Each child with UCP was age-matched with a TD child (mean age 9 years 4 months; SD = 2 years 1 month), recruited from Belgian schools, to avoid bias linked to age-effect.

This study was approved by the ethical committee of the Université catholique de Louvain. Children and caregivers provided their written informed consent to participate in the study.

### Experimental setup

**Fig 1:**
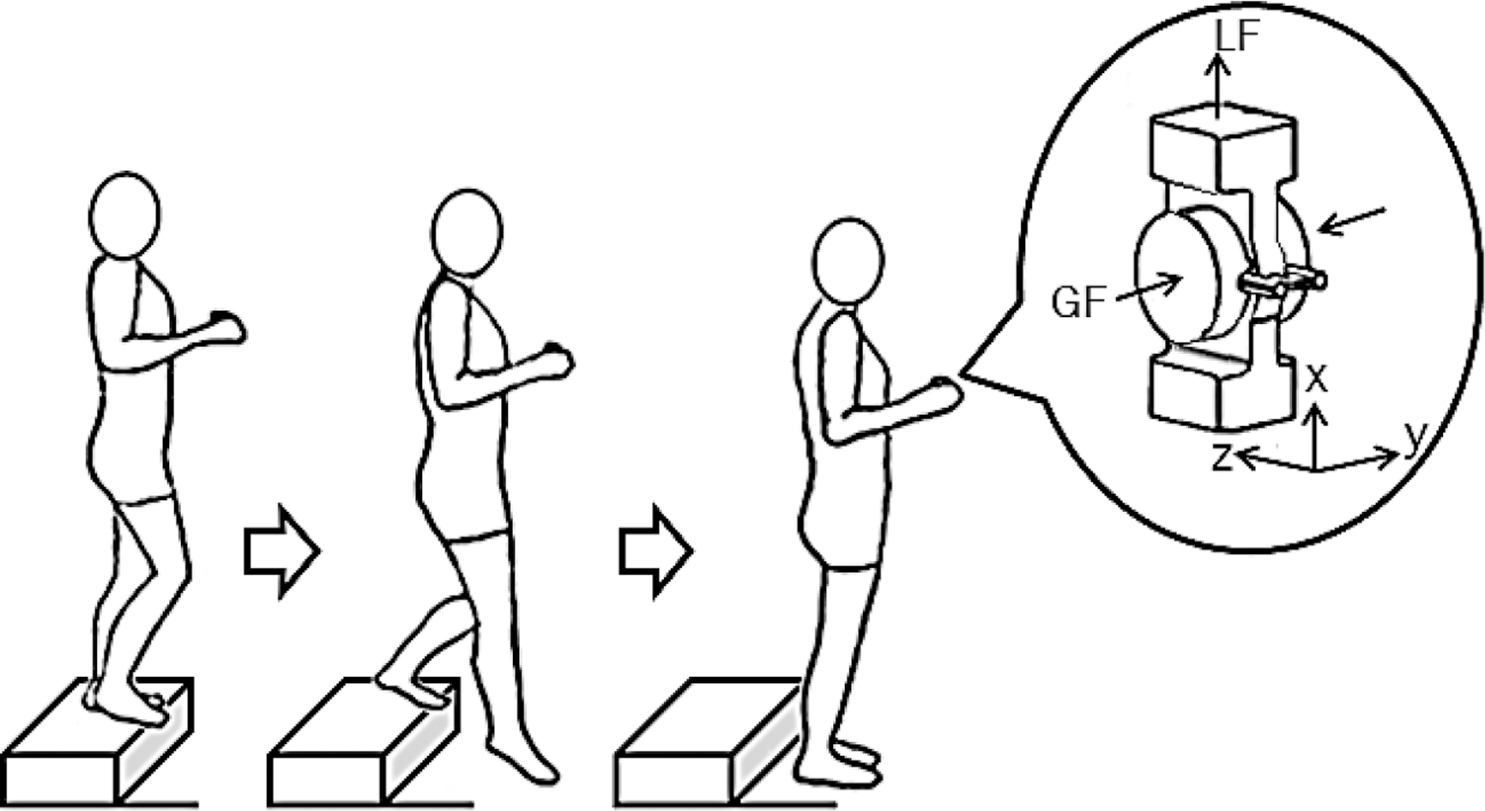
Diagram of a child holding the Manipulandum while performing the task from initial to ending position. LF, load force; GF, grip force. X, Y, and Z represent the vertical, mediolateral, and anteroposterior axes, respectively.

The instruments included a wooden step (16.5×61×30 cm), and a grip-lift Manipulandum [25] (Arsalis) with a mass of 220 g and width of 30 mm (Fig 1). The Manipulandum was used to measure the grip force (GF, perpendicular to the object in hand) and the load force (LF, tangential to the object in hand). The calculations were made based on three orthogonal force components (Fx, Fy, and Fz) measured by two (left and right) three-dimensional mini-40 force and torque sensors (ATI Industrial Automation, Apex, NC, USA). The Fx, Fy, and Fz sensing ranges were 40, 40, and 120 N, with 0.002, 0.002, and 0.006 N resolutions, respectively. LF was calculated as LF = LF_right_ + LF_left_, where LF_i_ $=\sqrt{F_{x}^{2}+F_{z}^{2}}$ for each sensor (i = right, left). GF was calculated as $\mathrm{GF}=\frac{F_{y,r}-F_{y,l}}{2}$, where r and l correspond to the right and left sensors, respectively. The signals from the sensors were acquired at a sampling frequency of 800 Hz and were digitized online using the Grip-lift Manipulandum-Box signal conditioner (Arsalis, Belgium). The signal data were registered and transferred to a portable computer allowing for later offline analysis.

### Procedure and experimental protocol

Before beginning the experiment, the children were asked to wash and dry their hands. Next, the task was explained to each child. While standing on the step, each child was asked to grasp the Manipulandum with precision grip (Fig 1). The child held the Manipulandum upright and oriented forward with the elbow flexed at 90°. Then the child was asked to go down the step in a spontaneous manner and to maintain a static bipedal ending position. The task was performed five times with each hand, starting always with the less affected/dominant hand. Each child performed 10 trials.

In addition, the maximum voluntary contraction (MVC) of the GF exerted was measured in a static standing position. At the end of the acquisition procedure, each child grasped the Manipulandum with his or her elbow flexed at 90° as hard as possible for 3 to 5 seconds with each hand. The mean MVC was calculated from three trials.

### Acquisition and data analysis

**Fig 2:**
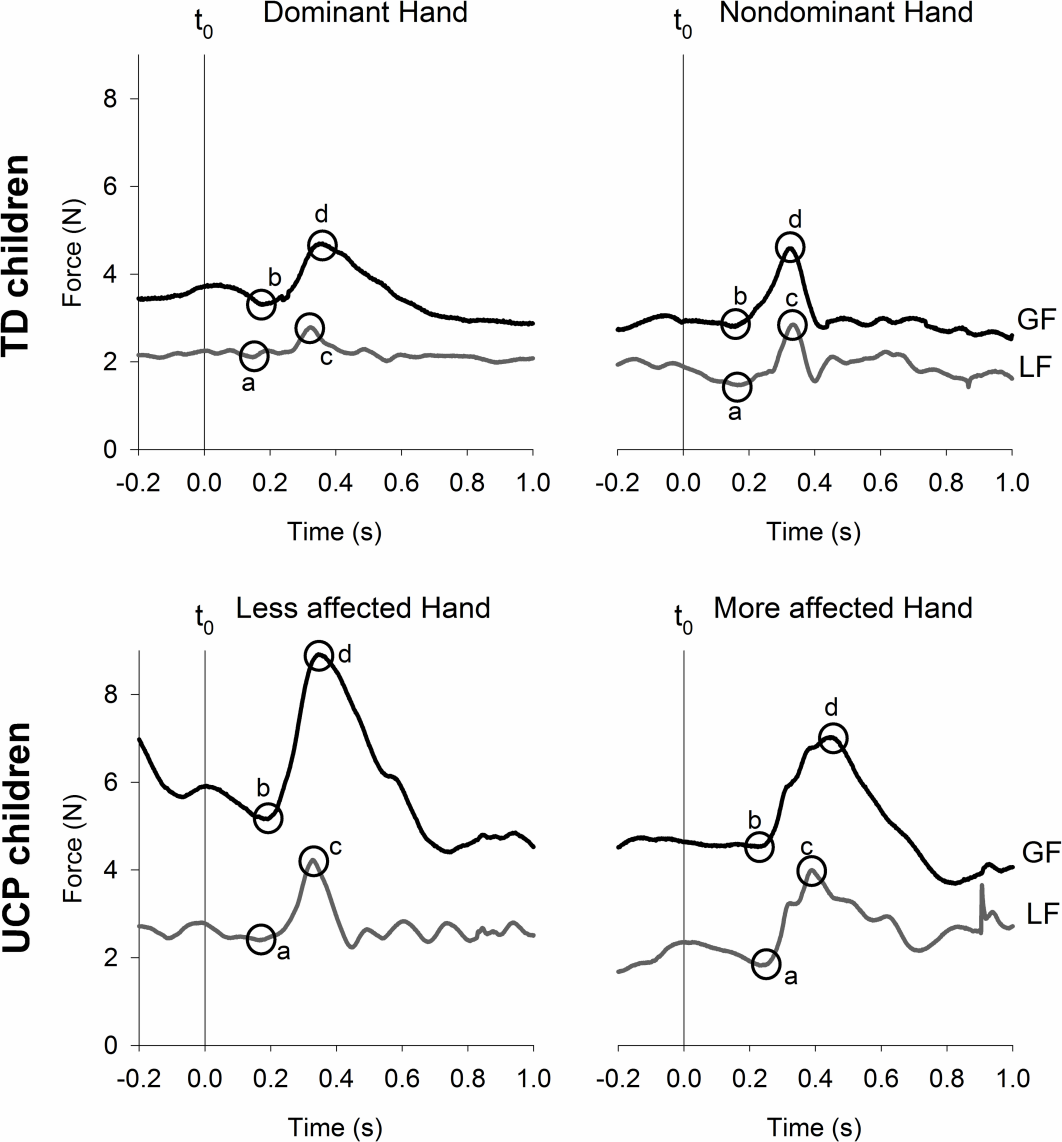
Representative traces of typically developing (TD) children and children with unilateral cerebral palsy (UCP) during one trial. The grip force (GF, black) and load force (LF, grey) are presented as a function of time in seconds (s) and measured in Newton (N). t_0_ = vertical line, start of child’s downward movement. The circles highlights the events observed in the forces during the task: a = minimal load force value observed during the task (LF_min_), b = minimal grip force value observed during the task (GF_min_), c = maximal load force value observed during the task (LF_max_), d = maximal grip force value observed during the task (GF_max_).

The temporal course of the task is displayed in Fig 2. We determined the t_0_ moment when the LF value just passed under the weight of the Manipulandum, indicating the start of the downward movement (Fig 2, vertical line). After t_0_, the LF and GF decreased during the downward movement, reaching the minimal load force (LF_min_) and minimal grip force (GF_min_) (Fig 2, circles labeled a and b). Then, the LF and GF started to increase, reaching the maximal load force (LF_max_) and maximal grip force (GF_max_) (Fig 2, circles labeled c and d). After these peaks, the LF and GF decreased. From these four temporal events (LF_min_, GF_min_, LF_max_, and GF_max_), we defined the following four temporal variables: LF_min_ to GF_min_ (Fig 2A and 2B), LF_min_ to LF_max_ (Fig 2A–2C), GF_min_ to LF_max_ (Fig 2B and 2C) and LF_max_ to GF_max_ (Fig 2C and 2D). The GF and LF amplitudes were also measured at the four events, resulting in eight dynamic variables. Furthermore, the ability to scale the GF to the LF (GF/LF ratio) was also calculated at each event. Data analysis was performed using MATLAB R2007b to avoid human bias.

### Statistics

A one-way repeated measures analysis of variance (ANOVA_RM_) was used across trials to test whether a training effect was observed on the dependent variables. As no training effect was observed (all *p*>0.05), the subsequent analyses were conducted considering the mean values of all trials for each subject (n = 50). An ANOVA_RM_ was also conducted to compare the four conditions, namely, the more affected hand and less affected hand of children with UCP and the nondominant hand and dominant hand of the controls. The post-hoc analysis was performed using Holm-Sidak pairwise comparisons between the conditions. An adjustment of the alpha level of significance was performed for pairwise multiple comparisons.

## Results

Representative traces for both hands of the TD children and children with UCP are displayed in Fig 2 (single trials). After t_0_, the LF and the GF reached their minimum values, with the LF_min_ (Fig 2A) occurring first followed by the GF_min_ (Fig 2B). Consecutively, the forces reached their maximum values, with the LF_max_ first followed by the GF_max_ (Fig 2C and 2D, respectively). In children with UCP, the less affected hand presented a temporal course similar to that of TD children (Fig 2A–2D). In contrast, the more affected hand presented a temporal shift in the force minima: the GF reached its minimum (Fig 2B) before the LF (Fig 2A). In the representative traces, the GF and LF were higher for both hands of the children with UCP compared to the TD children.

Statistical comparisons of the four conditions (more affected hand, less affected hand, nondominant hand, and dominant hand) were performed for the different variables. We systematically performed the following post-hoc analyses and compared both hands of children with UCP (more affected vs. less affected), both hands of TD children (nondominant vs. dominant), nondominant hands between the two groups of children (more affected vs. nondominant), and dominant hands between the two groups (less affected vs. dominant).

**Fig 3:**
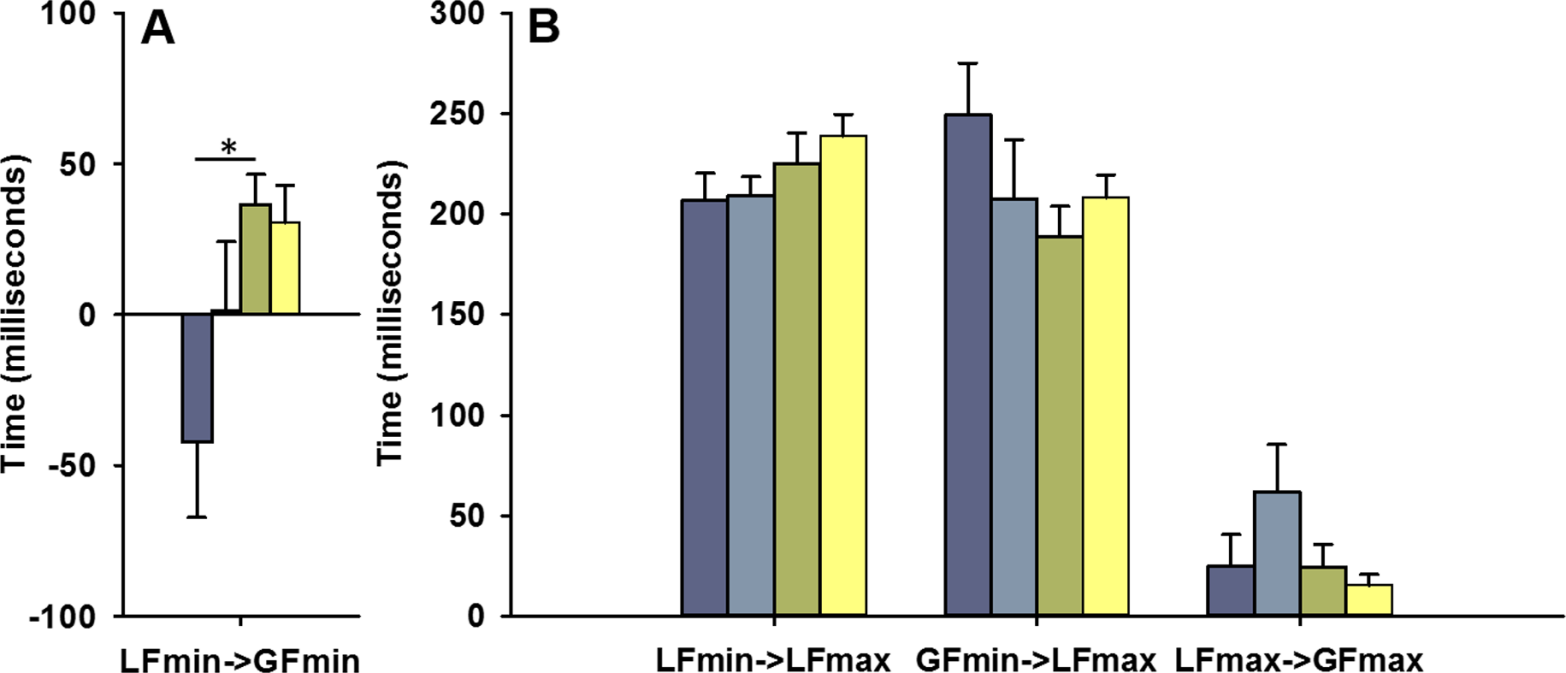
Results of temporal variables. A) Mean delay between LF_min_ and GF_min_ events. B) Mean delay between events of minima and maxima of forces. The four conditions are presented in colour code as follow: blue = more affected hand, cyan = less affected hand, green = nondominant hand and yellow = dominant hand. Error bars represent the standard error. * indicates p-value <0.050.

For the temporal variables, a difference between conditions was only observed in the delay between LF_min_ and GF_min_ (*p =* 0.038, see Table 1). Post-hoc analysis showed differences in this delay between the more affected hand of children with UCP and the nondominant hand of TD children (*p =* 0.009, Fig 3A), where the more affected hand showed negative mean values. This result indicates a shift in the temporal course of the more affected hand in children with UCP because the GF reached its minimum before the LF (Fig 2).

**Table 1:** Mean values of temporal and dynamic variables.

VARIABLES	MAH	LAH	NDH	DH	ANOVA_RM_	Post-hoc analyses, *p*-values			
	Mean (SD)	Mean (SD)	Mean (SD)	Mean (SD)	*p*-values	MAH vs LAH	NDH vs DH	MAH vs NDH	LAH vs DH
***Temporal variables***									
**LF**_**min**_**→GF**_**min**_ **(ms)**	-42.37 (108.6)	1.42 (114.3)	36.58 (48.6)	30.55 (61.2)	**0.038**	0.186	0.766	**0.009**	0.234
**LF**_**min**_**→LF**_**max**_ **(ms)**	206.70 (58.7)	209.00 (48.4)	225.12 (74.4)	238.71 (54.0)	0.238				
**GF**_**min**_**→LF**_**max**_ **(ms)**	249.07 (113.2)	207.58 (147.5)	188.54 (74.4)	208.16 (56.5)	0.458				
**LF**_**max**_**→GF**_**max**_ **(ms)**	24.93 (68.5)	61.60 (119.2)	24.46 (55.7)	15.31 (27.4)	0.172				
***Dynamic variables***									
**LF**									
**LF**_**min**_ **(N)**	1.64 (0.7)	2.22 (0.3)	1.61 (0.1)	1.64 (0.2)	**<0.001**	**<0.001**	0.877	0.837	**<0.001**
**LF at GF**_**min**_ **(N)**	2.04 (0.8)	2.49 (0.4)	1.73 (0.1)	1.76 (0.2)	**<0.001**	**0.002**	0.845	0.019^a^	**<0.001**
**LF**_**max**_ **(N)**	3.38 (1.1)	3.66 (0.6)	2.30 (0.3)	2.29 (0.3)	**<0.001**	0.106	0.966	**<0.001**	**<0.001**
**LF at GF**_**max**_ **(N)**	2.60 (0.8)	3.33 (0.5)	2.13 (0.3)	2.12 (0.2)	**<0.001**	**<0.001**	0.927	**0.003**	**<0.001**
**GF**									
**GF at LF**_**min**_ **(N)**	7.13 (7.6)	8.62 (9.7)	4.15 (1.9)	4.73 (1.9)	**0.025**	0.390	0.677	0.097	0.019^a^
**GF**_**min**_ **(N)**	5.95 (6.6)	7.64 (8.8)	3.89 (1.7)	4.45 (1.8)	0.053				
**GF at LF**_**max**_ **(N)**	8.01 (7.6)	10.12 (10.8)	4.39 (1.7)	5.23 (1.8)	**0.005**	0.278	0.593	0.055	**0.006**
**GF**_**max**_ **(N)**	8.92 (7.9)	11.06 (11.7)	4.59 (1.8)	5.40 (1.9)	**0.002**	0.310	0.620	0.032^a^	**0.003**
**GF/LF ratio**									
**GF/LF at LF**_**min**_ **(N)**	6.20 (3.6)	3.84 (3.7)	2.59 (1.1)	2.93 (1.1)	**<0.001**	**0.003**	0.588	**<0.001**	0.210
**GF/LF at GF**_**min**_ **(N)**	3.66 (2.3)	2.80 (2.4)	2.26 (1.0)	2.55 (0.9)	0.313				
**GF/LF at LF**_**max**_ **(N)**	2.44 (1.4)	2.62 (2.2)	1.95 (0.7)	2.27 (0.7)	0.071				
**GF/LF at GF**_**max**_ **(N)**	3.71 (2.0)	3.37 (3.1)	2.21 (0.8)	2.55 (0.8)	**0.025**	0.510	0.471	0.009^a^	0.124

MAH, more affected hand; LAH, less affected hand; NDH, nondominant hand; DH, dominant hand; LF, load force; GF, grip force; ms, milliseconds; N, Newtons. Significant differences are presented in bold

a, nonsignificant difference when the alpha level was adjusted using the Holm-Sidak multiple comparison method.

**Fig 4:**
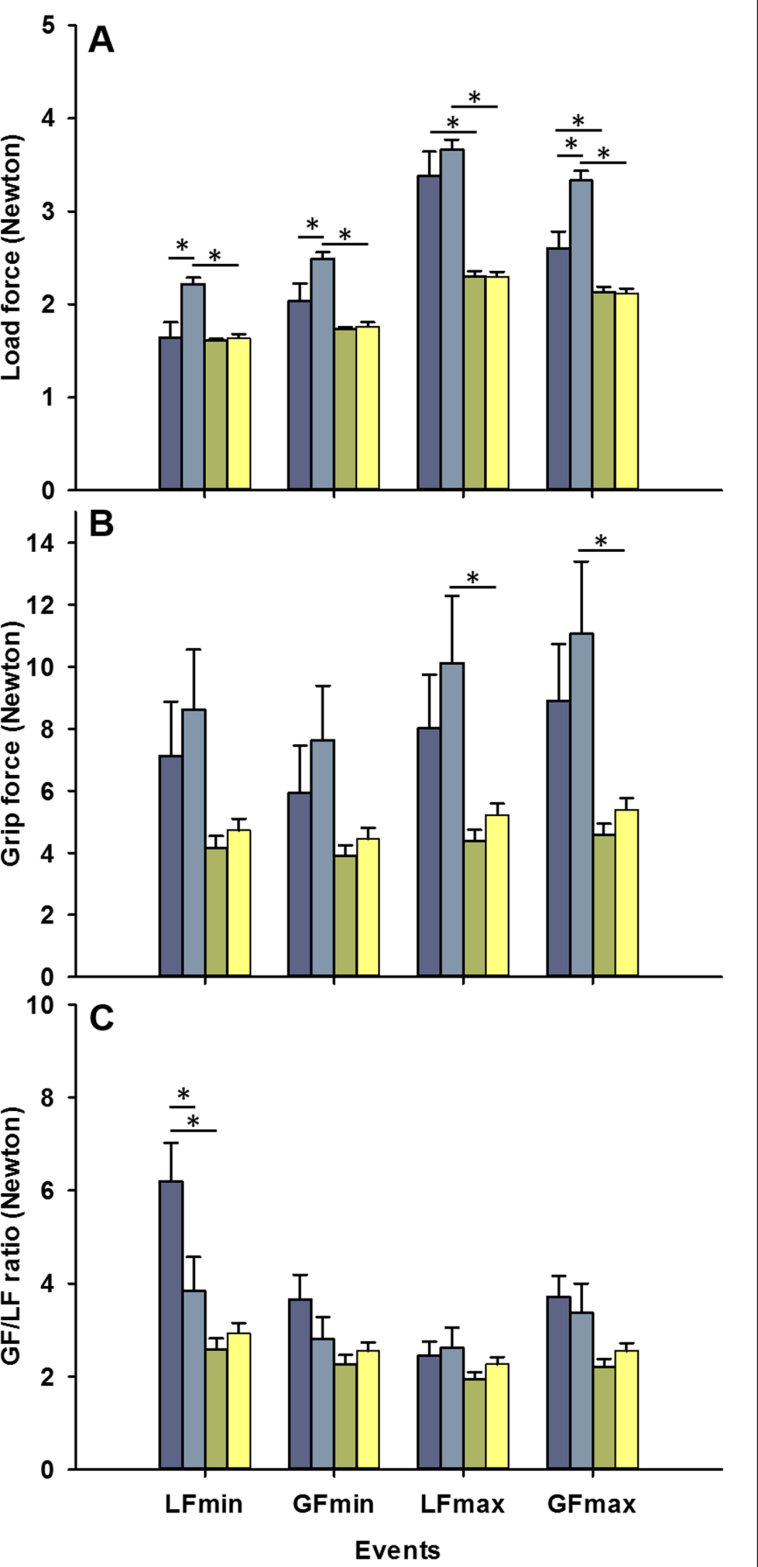
Results of dynamic variables in function of the events (LF_min_, GF_min_, LF_max_ and GF_max_). A) Mean Load force. B) Mean Grip force. C) Mean GF/LF ratio. The four conditions are presented in colour code as follow: blue = more affected hand, cyan = less affected hand, green = nondominant hand and yellow = dominant hand. Error bars represent the standard error. * indicates p-value <0.050.

Differences were observed in the LF amplitudes among the conditions at the different temporal events (all *p*<0.001, Table 1). Post-hoc comparisons showed that children with UCP had systematically higher LF values on the less affected hand when compared to the dominant hand of controls (all *p*<0.001, see Fig 4A). Higher LF values were also observed on the more affected hand of children with UCP when compared to the nondominant of controls, with significant differences at the LF_max_ and GF_max_. Finally, a significant difference was noted in LF amplitudes between the two hands of children with UCP at almost all events, with higher LF levels for the less affected hand than for the more affected. No difference was observed in the LF values between the two hands of the TD children (all *p*≥0.845).

In addition, differences were observed for the GF amplitude among the conditions for almost all of the temporal events (all *p*≤0.025), except for the GF_min_ (*p =* 0.053; Table 1). Post-hoc comparisons showed that children with UCP presented higher GF values on the less affected hand when compared to the dominant hand of controls at LF_max_ and at GF_max_ (see Fig 4B). The children with UCP also presented higher GF values on the more affected hand when compared to the nondominant of controls, although these differences were not significant after the alpha level was adjusted (Table 1). No differences were observed in the GF amplitudes between the two hands of children with UCP (all *p*≥0.278) or between the two hands of TD children (all *p*≥0.593).

A difference was observed in the GF/LF ratio among the conditions at LF_min_ (*p*<0.001) and GF_max_ (*p =* 0.025). Post-hoc comparisons showed higher GF/LF ratios at LF_min_ for the more affected hand of children with UCP when compared to either the nondominant hand of controls (*p*<0.001, see Fig 4C) or the less affected hand (*p =* 0.003). We found no significant difference among the conditions at GF_max_ in the post-hoc analysis after the alpha level of significance was adjusted.

We observed differences in the MVC among the conditions (*p*<0.001, Table 2). Post-hoc comparisons showed that children with UCP had systematically lower MVC values for the more affected hand when compared to the nondominant of controls or the less affected hand (all *p*<0.001).

**Table 2:** Mean values of maximum voluntary contraction and percentage of maximum voluntary contraction for the grip force.

VARIABLES	MAH	LAH	NDH	DH	ANOVA_RM_	Post hoc analyses, *p*-values			
	Mean (SD)	Mean (SD)	Mean (SD)	Mean (SD)	*p*-values	MAH vs LAH	NDH vs DH	MAH vs NDH	LAH vs DH
**MVC in static condition (N)**	16.73 (7.6)	40.35 (21.7)	37.70 (16.0)	43.19 (15.6)	**<0.001**	**<0.001**	0.208	**<0.001**	0.276
**% of MVC for GF**									
**GF at LF**_**min**_ **(%)**	44.61 (34.3)	23.99 (32.1)	12.73 (7.3)	12.61 (7.3)	**<0.001**	**0.006**	0.985	**<0.001**	0.065
**GF**_**min**_ **(%)**	37.32 (29.2)	21.64 (29.4)	12.0 (7.0)	11.93 (7.2)	**<0.001**	0.017^a^	0.990	**<0.001**	0.082
**GF at LF**_**max**_ **(%)**	50.79 (33.9)	28.0 (36.1)	13.54 (7.2)	13.79 (7.5)	**<0.001**	**0.004**	0.971	**<0.001**	0.026^a^
**GF**_**max**_ **(%)**	56.33 (35.1)	30.20 (38.6)	14.09 (7.3)	14.19 (7.5)	**<0.001**	**0.002**	0.989	**<0.001**	**0.018**

MAH, more affected hand; LAH, less affected hand; NDH, nondominant hand; DH, dominant hand; MVC, maximum voluntary contraction; LF, load force; GF, grip force; N, newton. Significant differences are presented in bold

a, nonsignificant difference when the alpha level was adjusted using the Holm-Sidak multiple comparison method.

Systematic differences were observed among the conditions in the percentage of MVC (%MVC) used for GF for all of the temporal events (*p*<0.001). Post-hoc comparisons showed that children with UCP had systematically higher %MVC values for GF for the more affected hand when compared to the nondominant hand of controls (all *p*<0.001). Children with UCP also presented higher %MVC values for the less affected hand when compared to the dominant hand of controls at GF_max_. Finally, there were differences between the two hands of children with UCP at almost all events, with a higher %MVC in the more affected hand (all *p*≤0.017).

## Discussion

In this study, we aimed to assess the motor control of precision grip in children with UCP while they walked down a step. Our hypothesis that children with UCP would show impaired anticipation control was partially supported because the temporal course was only perturbed for the more affected hand at the start of the task. Higher LF values were observed for all temporal events in both hands of children with UCP compared to the controls. Higher GF values were also observed in the less affected hand during the maximum load increase in children with UCP, although the GF/LF ratio was similar to controls. Conversely, in the more affected hand, the GF/LF ratio was higher than in the controls at the onset of the load increase. Furthermore, in the more affected hand, children with UCP had lower MVCs leading to a higher %MVC during the task than the other conditions.

In this study, the TD children presented an early coupling of the GF and LF while walking down the step. Similarly to adults [25], during the initial downward movement of the body from the step, children first showed a parallel decrease in the forces to their minima. Subsequently, an increase in the LF was followed shortly by an increase in the GF, which corresponded to an upward movement of the arm [25]. This early coupling was present but displayed a shift in the more affected hand of children with UCP; the GF increase was observed before the LF increase. This temporal perturbation may rely on processes similar to those involved at the start of a grip-lift task. The preload phase, which is the time between the GF and LF onset, was increased in children with UCP when compared to TD children [14]. This result was interpreted as a strategy to integrate more tactile feedback to adapt the GF and avoid slips [14].

From their minima, the GF and LF increased progressively in parallel, anticipating the brisk force change during the maximal charge of the body weight (at LF_max_), the moment when the object is most likely to slip. The anticipatory increases in LF and GF started at 207.9ms (mean time in children with UCP) and 231.9ms (mean time for TD children) before LF_max_. These values closely match previous observations in adults performing the same task (approximately 220ms [25]). In tasks performed in a seated position, comparable delays have been described in predictive control of GF/LF during brisk load increases induced directly on a handheld object, with GF peaks arising 280ms after the brisk load increase [6]. Thus, it seems that brisk load changes induced by the lower extremities are integrated and anticipated in similar ways to those induced directly on the object. In both experiments, children with UCP have the ability to adapt the anticipatory timing adequately.

Children with UCP presented higher LF and GF values than the TD children. These results are in agreement with previous studies of grip-lift tasks in children with UCP [4]. The LF increased in both hands of children with UCP, suggesting that there were larger vertical displacements of the handheld object [6, 25]. In the less affected hand, the GF increased as an efficient adaptation to the higher LF as the GF/LF ratio was similar to that of controls. In the more affected hand, this ratio was also similar to controls except at LF_min_ (descent from the step), when GF was overestimated leading to a higher GF/LF ratio. The higher GF values in children with UCP may be an adaptation to generate GF/LF ratios similar to TD children, suggesting that there is anticipatory control in both hands [20, 21]. However, the overestimation of GF at LF_min_ may be explained by less modulation of the forces and a calibration based on the maximal constraint. This overestimation of GF has been demonstrated previously in grip-lift tasks [14, 15] and is proposed to compensate for a deficit in feedback integration in the anticipatory control of a task. This anticipatory control has been associated with internal modelling [29], which generates a prediction of the action based on the sensory information previously stored (e.g., proprioception) and on the efference copy of a motor command allowing for movement correction [30].

A significantly lower MVC was observed for the more affected hand of children with UCP compared with the less affected and with both hands of TD children. Nevertheless, children with UCP developed higher GF values, leading to higher %MVCs in the more affected hand. At LF_max_, over 50% of the MVC was used in the more affected hand, while less than 14% of the MVC was developed in both hands of TD children. This high %MVC likely tires children with UCP. In the literature, the use of a 20% MVC is described as a low intensity contraction in hand muscles, whereas an MVC over 50% is considered to be a high intensity contraction [31]. Previously, the use of a high %MVC (over 50%) has been reported for the affected hands of children with CP in low-load tasks [14, 20, 32]. These observations on the %MVC show that for the more affected hand, children with UCP have a double disadvantage. First, they have difficulty in scaling the forces, probably due to a lack of feedback integration and a higher GF than needed for the task. Second, they have a lower MVC. These alterations may lead to early fatigue and an inability to transport objects in optimal or comfortable manners.

The present study aimed to observe the fingertip force coordination during a discrete task of walking down a step, which is very common in daily life e.g., step off the sidewalk while carrying an object. Unexpectedly, regarding previous results observed during walking while carrying an object, we did not find a desynchronization of the peak of the forces in the more affected hand of children with UCP [21]. Indeed, while walking, Prabhu et al. observed that children with UCP presented impairments in fingertip forces control in the more affected hand resulting in a desynchronization of GF and LF at their peaks, shortly after the contact of the foot on the ground, at the peak of gait’s perturbation. They proposed that a lateralized sensorimotor integration impairment underlies the altered synchronization observed in their results. In our study, though subtle timing differences were observed at the onset of LF increase in the more affected hand, the timing of forces was preserved at the forces peak, during the maximum increase in load. The difference between our results and those of Prabhu et al. may be related, at least in part, to the different neural basis while performing cyclic or discrete movements [33, 34]. Discrete movements require more cortical and subcortical involvement than cyclic movements: in addition to the primary motor areas–active during cyclic movements–the planning and performance of discrete tasks require the activation of prefrontal and parietal areas as well as the cerebellum [33–35]. It is thus likely that the different control loops of the movement–either discrete or cyclic–are affected in a different way during upper and lower extremity coordination in children with UCP, cyclic tasks presenting more timing impairments. This is in line with previous observations on the coordination pattern of upper and lower extremity during gait without object manipulation: the cyclic arm swing accompanying gait is disrupted in children with UCP while walking at self-selected speed [36]. It is also likely that the manipulation with the more affected hand in our discrete task have been advantaged by a bilateral transfer: the task was performed first with the less affected hand and then with the more affected hand which may have allowed a positive effector-specificity skill transfer [21].

However, though the discrete task used here was unexpectedly well timed, like in cyclic movements, this task highlighted specific deficits lateralized in the more affected upper extremity, reinforcing the hypothesis of a lateralized sensorimotor integration impairment underlying the altered coordination of the forces.

Differences in the load forces might be due to an altered upper and lower extremity coordination like that observed during gait in children with UCP [21]. However, though an increase in the GL/LF ratio was observed while walking compared to a standing position, no difference in LF was reported in the more or less affected hand [21]. These results suggest no major alteration on forces during gait with object transport and are thus not likely the basis of the results observed here. However, data from gait with object transport also demonstrated strategy differences between the more and the less affected hand. The authors suggested an overall increase of the GF/LF ratio related to a less ability to adapt the GF in the more affected hand only, with a lateralized deficit in movement planning [21]. This suggestion is congruent with a lack of update of motor command due to alerted tactile/proprioceptive feedbacks in the more affected hand [18, 20, 37]. Increases in ratios in the present study may actually reflect the attempts of children to aid grip control regarding their diminished ability to integrate the different components of movement. The higher LF observed during the task might be related either to a larger arm movement during the task or to a stronger impact while going down the step.

Our results contribute to a better understanding of the precision grip forces in dynamic conditions, matching everyday life requirements. Specifically, these results provide more insights regarding discrete tasks involving the coordination of upper and lower extremities, different from the gait but also very common in daily life. The ability to manage the task, but the high amount of forces needed in children with UCP, suggests that the coordination of upper and lower extremities might be of interest to introduce in rehabilitation programs dedicated to these children. A recent intensive rehabilitation process has focused on this coordination and has demonstrated to be useful for improving both upper and lower extremities abilities [28, 38]. A coordination task, like the stair step task proposed in this study, might also be used to assess changes in the upper/lower extremities coordination before and after such interventions to define if the coordination per se is modified by the training.

### Limitations

Although the children in this study were age-matched, gender, weight, or height may have an effect on the regulation of fingertip forces during the task. This study focused on transport of a handheld object with one hand or the other. In everyday life, it is likely that children with UCP favor transporting objects using both hands. The coordination of both hands during such a task was not measured in this study.

## Conclusion

Children with UCP presented higher load and grip forces on both hands. However, the GF was well scaled to the LF for both hands, except for the more affected hand at the start of the task when an overestimation was observed. These findings highlight an anticipatory control in children with UCP, which is adequate on the less affected hand but slightly altered on the more affected hand, probably due to a deficit in feedback integration. In addition, a decreased ability to develop forces in the more affected hand was observed. This altered force scaling and force generation may lead to early fatigue and consequently limit daily life activities. Regarding the specific strategies developed by children with UCP to manage a task requiring the coordination of upper and lower extremities–notably the use of a high force percentage–we suggest that these children might benefit from therapies targeting this coordination. A potential training allowing the use of a lower percentage of force might have a positive impact on the activity level and the autonomy of these children since these coordination tasks are very common in everyday life.

## References

[1] Graham HK, Rosenbaum P, Paneth N, Dan B, Lin JP, Damiano DL, et al Cerebral palsy. Nature reviews Disease primers. 2016;2:15082 Epub 2016/05/18. doi: 10.1038/nrdp.2015.82 . 27188686 10.1038/nrdp.2015.82 PMC9619297

[2] Uvebrant P. Hemiplegic cerebral palsy. Aetiology and outcome. Acta Paediatr Scand Suppl. 1988;345:1–100. . 3201989 10.1111/j.1651-2227.1988.tb14939.x

[3] Eliasson AC, Forssberg H, Ikuta K, Apel I, Westling G, Johansson R. Development of human precision grip. V. anticipatory and triggered grip actions during sudden loading. Exp Brain Res. 1995;106(3):425–33. Epub 1995/01/01. . 8983986 10.1007/BF00231065

[4] Forssberg H, Eliasson AC, Redon-Zouitenn C, Mercuri E, Dubowitz L. Impaired grip-lift synergy in children with unilateral brain lesions. Brain a journal of neurology. 1999;122 Pt 6):1157–68. Epub 1999/06/04. . 10356067 10.1093/brain/122.6.1157

[5] Forssberg H, Eliasson A-C, Kinoshita H, Johansson RS, Westling G. Development of human precision grip. I: Basic coordination of force. Exp Brain Res. 1991;85(2):451–7. . 1893993 10.1007/BF00229422

[6] Bleyenheuft Y, Lefevre P, Thonnard JL. Predictive mechanisms control grip force after impact in self-triggered perturbations. J Mot Behav. 2009;41(5):411–7. doi: 10.3200/35-08-084 . 19460751 10.3200/35-08-084

[7] Blakemore SJ, Goodbody SJ, Wolpert DM. Predicting the consequences of our own actions: the role of sensorimotor context estimation. J Neurosci. 1998;18(18):7511–8. . 9736669 10.1523/JNEUROSCI.18-18-07511.1998 PMC6793221

[8] Flanagan JR, Wing AM. The role of internal models in motion planning and control: evidence from grip force adjustments during movements of hand-held loads. J Neurosci. 1997;17(4):1519–28. . 9006993 10.1523/JNEUROSCI.17-04-01519.1997 PMC6793733

[9] Witney AG, Wing A, Thonnard JL, Smith AM. The cutaneous contribution to adaptive precision grip. Trends Neurosci. 2004;27(10):637–43. doi: 10.1016/j.tins.2004.08.006 . 15374677 10.1016/j.tins.2004.08.006

[10] Eliasson AC, Forssberg H, Hung YC, Gordon AM. Development of hand function and precision grip control in individuals with cerebral palsy: a 13-year follow-up study. Pediatrics. 2006;118(4):e1226–36. doi: 10.1542/peds.2005-2768 . 17015511 10.1542/peds.2005-2768

[11] Gordon AM, Forssberg H, Johansson RS, Westling G. Visual size cues in the programming of manipulative forces during precision grip. Exp Brain Res. 1991;83(3):477–82. . 2026190 10.1007/BF00229824

[12] Johansson RS, Westling G. Coordinated isometric muscle commands adequately and erroneously programmed for the weight during lifting task with precision grip. Exp Brain Res. 1988;71(1):59–71. . 3416958 10.1007/BF00247522

[13] Westling G, Johansson RS. Factors influencing the force control during precision grip. Exp Brain Res. 1984;53(2):277–84. . 6705863 10.1007/BF00238156

[14] Eliasson AC, Gordon AM, Forssberg H. Basic co-ordination of manipulative forces of children with cerebral palsy. Dev Med Child Neurol. 1991;33(8):661–70. . 1916022 10.1111/j.1469-8749.1991.tb14943.x

[15] Eliasson AC, Gordon AM, Forssberg H. Impaired anticipatory control of isometric forces during grasping by children with cerebral palsy. Developmental medicine and child neurology. 1992;34(3):216–25. Epub 1992/03/01. . 1559601 10.1111/j.1469-8749.1992.tb14994.x

[16] Gordon AM, Duff SV. Fingertip forces during object manipulation in children with hemiplegic cerebral palsy. I: anticipatory scaling. Dev Med Child Neurol. 1999;41(3):166–75. . 10210249 10.1017/s0012162299000353

[17] Steenbergen B, Hulstijn W, Lemmens IH, Meulenbroek RG. The timing of prehensile movements in subjects with cerebral palsy. Dev Med Child Neurol. 1998;40(2):108–14. Epub 1998/03/07. . 9489499

[18] Gordon AM, Charles J, Steenbergen B. Fingertip force planning during grasp is disrupted by impaired sensorimotor integration in children with hemiplegic cerebral palsy. Pediatr Res. 2006;60(5):587–91. doi: 10.1203/01.pdr.0000242370.41469.74 . 16988186 10.1203/01.pdr.0000242370.41469.74

[19] Steenbergen B, Charles J, Gordon AM. Fingertip force control during bimanual object lifting in hemiplegic cerebral palsy. Experimental brain research. 2008;186(2):191–201. Epub 2008/01/29. doi: 10.1007/s00221-007-1223-6 ; PubMed Central PMCID: PMCPmc2668615. 18224309 10.1007/s00221-007-1223-6 PMC2668615

[20] Bleyenheuft Y, Thonnard JL. Predictive and reactive control of precision grip in children with congenital hemiplegia. Neurorehabilitation and neural repair. 2010;24(4):318–27. Epub 2009/12/05. doi: 10.1177/1545968309353327 . 19959831 10.1177/1545968309353327

[21] Prabhu SB, Diermayr G, Gysin P, Gordon AM. Coordination of fingertip forces in object transport during gait in children with hemiplegic cerebral palsy. Dev Med Child Neurol. 2011;53(9):865–9. doi: 10.1111/j.1469-8749.2011.04061.x . 21790557 10.1111/j.1469-8749.2011.04061.x

[22] Gysin P, Kaminski TR, Gordon AM. Dynamic Grasp Control during Gait In: Hermsdœrfer J, Nowak DA, editors. Sensorimotor control of grasping physiology and pathophysiology. Cambridge New York: Cambridge University Press,; 2009 p. xiv, 509 p., 12 p. of plates ill. (some col.).

[23] Gysin P, Kaminski TR, Hass CJ, Grobet CE, Gordon AM. Effects of gait variations on grip force coordination during object transport. J Neurophysiol. 2008;100(5):2477–85. doi: 10.1152/jn.90561.2008 . 18753327 10.1152/jn.90561.2008

[24] Bernshteĭn NA. The co-ordination and regulation of movements. Oxford: Pergamon; 1967 xii, 196 p p.

[25] Ebner-Karestinos D, Thonnard JL, Bleyenheuft Y. Precision Grip Control while Walking Down a Stair Step. PLoS One. 2016;11(11):e0165549 doi: 10.1371/journal.pone.0165549 . 27802343 10.1371/journal.pone.0165549 PMC5089719

[26] Eliasson AC, Krumlinde-Sundholm L, Rosblad B, Beckung E, Arner M, Ohrvall AM, et al The Manual Ability Classification System (MACS) for children with cerebral palsy: scale development and evidence of validity and reliability. Dev Med Child Neurol. 2006;48(7):549–54. doi: 10.1017/S0012162206001162 . 16780622 10.1017/S0012162206001162

[27] Palisano R, Rosenbaum P, Walter S, Russell D, Wood E, Galuppi B. Development and reliability of a system to classify gross motor function in children with cerebral palsy. Dev Med Child Neurol. 1997;39(4):214–23. . 9183258 10.1111/j.1469-8749.1997.tb07414.x

[28] Bleyenheuft Y, Arnould C, Brandao MB, Bleyenheuft C, Gordon AM. Hand and Arm Bimanual Intensive Therapy Including Lower Extremity (HABIT-ILE) in Children With Unilateral Spastic Cerebral Palsy: A Randomized Trial. Neurorehabil Neural Repair. 2015;29(7):645–57. doi: 10.1177/1545968314562109 . 25527487 10.1177/1545968314562109

[29] Wolpert DM. Computational approaches to motor control. Trends Cogn Sci. 1997;1(6):209–16. doi: 10.1016/S1364-6613(97)01070-X . 21223909 10.1016/S1364-6613(97)01070-X

[30] Steenbergen B, Jongbloed-Pereboom M, Spruijt S, Gordon AM. Impaired motor planning and motor imagery in children with unilateral spastic cerebral palsy: challenges for the future of pediatric rehabilitation. Dev Med Child Neurol. 2013;55 Suppl 4:43–6. doi: 10.1111/dmcn.12306 . 24237279 10.1111/dmcn.12306

[31] Maluf KS, Shinohara M, Stephenson JL, Enoka RM. Muscle activation and time to task failure differ with load type and contraction intensity for a human hand muscle. Experimental Brain Research. 2005;167(2):165–77. doi: 10.1007/s00221-005-0017-y 16044306 10.1007/s00221-005-0017-y

[32] Smits-Engelsman BC, Rameckers EA, Duysens J. Muscle force generation and force control of finger movements in children with spastic hemiplegia during isometric tasks. Dev Med Child Neurol. 2005;47(5):337–42. . 15892376 10.1017/s0012162205000630

[33] Howard IS, Ingram JN, Wolpert DM. Separate representations of dynamics in rhythmic and discrete movements: evidence from motor learning. J Neurophysiol. 2011;105(4):1722–31. doi: 10.1152/jn.00780.2010 ; PubMed Central PMCID: PMCPMC3075277. 21273324 10.1152/jn.00780.2010 PMC3075277

[34] Hogan N, Sternad D. On rhythmic and discrete movements: reflections, definitions and implications for motor control. Exp Brain Res. 2007;181(1):13–30. doi: 10.1007/s00221-007-0899-y . 17530234 10.1007/s00221-007-0899-y

[35] Schaal S, Sternad D, Osu R, Kawato M. Rhythmic arm movement is not discrete. Nat Neurosci. 2004;7(10):1136–43. doi: 10.1038/nn1322 . 15452580 10.1038/nn1322

[36] Meyns P, Van Gestel L, Massaad F, Desloovere K, Molenaers G, Duysens J. Arm swing during walking at different speeds in children with Cerebral Palsy and typically developing children. Res Dev Disabil. 2011;32(5):1957–64. doi: 10.1016/j.ridd.2011.03.029 . 21531534 10.1016/j.ridd.2011.03.029

[37] Gordon AM, Charles J, Duff SV. Fingertip forces during object manipulation in children with hemiplegic cerebral palsy. II: bilateral coordination. Dev Med Child Neurol. 1999;41(3):176–85. . 10210250 10.1017/s0012162299000365

[38] Bleyenheuft Y, Gordon AM. Hand-arm bimanual intensive therapy including lower extremities (HABIT-ILE) for children with cerebral palsy. Physical & occupational therapy in pediatrics. 2014;34(4):390–403. Epub 2014/10/02. doi: 10.3109/01942638.2014.932884 . 25271469 10.3109/01942638.2014.932884

